# Que te aproveche mirar lo que miras: Una reflexión humanista sobre la mirada paliativa

**DOI:** 10.23938/ASSN.1130

**Published:** 2025-09-26

**Authors:** Juan Luis Torres-Tenor

**Affiliations:** Hospital Universitario La Paz Unidad de Cuidados Paliativos Madrid España

**Keywords:** Palifobia, Paliativos, Narrativa, Significado, Holismo, Palliphobia, Palliative care, Narrative, Meaning, Holism

## Abstract

Este ensayo filosófico-literario reflexiona, desde la experiencia clínica de un médico especialista en cuidados paliativos, sobre la necesidad de resignificar esta disciplina dentro de la cultura sanitaria y social. A través de una mirada personal, con las letras del cantautor español Joaquín Sabina como vehículo conductor, se plantea que la resistencia institucional y emocional a los cuidados paliativos -la *palifobia*- no responde solo a barreras estructurales, sino también a una narrativa centrada exclusivamente en la curación. Se reivindica el papel esencial del personal sanitario en este cambio de paradigma, así como la importancia de recuperar una atención centrada en la persona, capaz de ver, escuchar y acompañar incluso cuando ya no es posible curar. Frente al ruido tecnológico y la prisa asistencial, el texto propone afinar la mirada para que, a ambos lados de la mesa de consulta, nos aproveche mirar lo que miramos.

## Abstract (en)

This essay, written from the clinical experience of a physician specialized in palliative care, reflects on the need to reframe this discipline within both medical practice and social culture. Through a personal lens, and using the lyrics of Spanish singer-songwriter Joaquín Sabina as a guiding thread, it argues that institutional and emotional resistance to palliative care - *palliphobia* - arises not only from structural barriers, but also from a medical narrative focused almost exclusively on cure. The text underscores the essential role of healthcare professionals in fostering this paradigm shift and the importance of reclaiming person-centered care: a way of seeing, listening, and accompanying, even when cure is no longer possible. Against the backdrop of technological noise and clinical haste, the essay proposes refining our gaze so that, on both sides of the consultation desk, we may rediscover meaning and value in what we see.

“Que el corazón no se pase de moda”

Joaquín Sabina*, Noches de boda* (1999)

De todas las virtudes del verdadero arte, hay tres que valoro especialmente. La primera es su capacidad para generar espacios comunes donde personas muy diferentes entre sí experimentan el milagro de los sentimientos compartidos. La segunda, la posibilidad de expresar la propia emoción y cognición mediante palabras, imágenes o sonidos de manera inaccesible para la mayoría. La tercera, la variabilidad de la interpretación personal, moldeada por el paso del tiempo, reflejo de la evolución de nuestro mundo interior. Siempre estuve de acuerdo con María Zambrano cuando escribió que *el arte es una forma de conocimiento más alta que la razón, porque llega más allá de lo que la razón puede alcanzar*^1^.

En la madrugada de un miércoles de mayo cualquiera, seguramente haya pensamientos más productivos, especialmente estando de guardia. Sin embargo, no puedo dejar de pensar en las verdades, mentiras y paradojas que se encuentran en el arte genuino, que obligan a replantearse la existencia, para bien o para mal. Supongo que mi desvelo tiene que ver con el primero de los conciertos madrileños de la gira de despedida de Joaquín Sabina, donde experimenté las tres bondades artísticas antes mencionadas: comunión, articulación de lo inefable y resonancia emocional.

Este cantante, uno de los hispanoparlantes más exitosos de las últimas décadas, no requiere presentación. Conocemos sus canciones y su habilidad para poetizar una amplia gama de afectos humanos, de ecléctico valor moral. Esta gira, anunciada como su última y titulada elocuentemente como *Hola y Adiós*, tiene un claro aroma a epílogo, a nostalgia, a cierre consciente de una larga trayectoria. Cual sesión grupal a gran escala de Psicoterapia Centrada en el Sentido^2^ o Terapia de la Dignidad^3^, el público adquiere conciencia de que una etapa llega a su ocaso, que esta fiesta no se repetirá. Y no me pareció, en absoluto, que la vivencia general fuera egodistónica. Siento que todos disfrutamos del sabor a despedida, del vinagre en las heridas; de ese pañuelo de estación^4^.

Un evento así contiene grandes dosis de arte, con la música y la poesía al frente. De las tres cualidades enumeradas, fue la tercera la que más sentí: la influencia de la perspectiva individual. Imagino que fui alcanzado por el concepto que expresó, infinitamente mejor que yo, Octavio Paz: *el poema es una obra abierta: cada lector lo recrea y lo transforma. La lectura es una operación activa, una participación*^5^. Tras años como médico especializado en cuidados paliativos, muchos versos de Sabina, así como multitud de canciones, películas, libros o pinturas, me evocan a quienes afrontan el final de la vida.

Pocas canciones conectan mejor con el público que *Noches de boda*^6^. Con versos que ya forman parte del imaginario colectivo, el cantante desea que triunfe lo verdaderamente valioso. El significado de esta obra se matiza con cada escucha, cada año vivido, cada cambio de nuestro mundo, tanto exterior como interior. No suena igual con la ingenuidad de la adolescencia, en su año de lanzamiento, a finales de los noventa, que *a mitad del camino de la vida*^7^, sentado en una consulta, con la sala de espera abarrotada. Todos anhelamos coincidir con lo que proclama esta ranchera mestiza, aunque aplicarlo no resulte tan sencillo. Nos encantaría priorizar lo importante frente a lo urgente. El corazón respecto al cerebro. Lo poético sobre lo prosaico.

Pero no es fácil. Muy difícil, de hecho, para quienes afrontan la crudeza de una enfermedad incurable avanzada. Todo profesional sanitario con cierta experiencia puede visualizar nítidamente unas cuantas personas para quienes ya ninguna noche será como una noche de boda, para quienes ninguna luna volverá a ser de miel. Por más que suene bien, ningún diccionario detiene las metastásicas balas disparadas por la prueba de imagen que muestra progresión. A veces, el desamparo se ocupa de todo. Las alas no soportan ciertos equipajes. El *puedo* gana bastantes guerras. Complicado que alguien aproveche mirar lo que mira, cuando solo mira el techo de la habitación de una unidad de cuidados paliativos. Allí, cuesta no contar las horas esperando un milagro, pues el calendario viene con prisa. Una cama de hospital tiñe pocas canas, no podemos engañarnos. El espejo del baño se compincha con la caquexia para darle la razón. La realidad, me temo, es que los otoños que preceden al último invierno no suelen dorar la piel^6^.

Visto así, el panorama se parece más a un desolado paisaje de antenas y de cables que a una pradera. Ni el campo está verde, ni es primavera^8^. En este contexto, resulta comprensible la tentación de intentar la curación hasta el final, incluso en situaciones en las que la evidencia sugiere su alta improbabilidad. *Que gane el quiero la guerra del puedo*^6^. Guerra que cada especialidad libra con sus mejores armas: oncología, con la inmunoterapia; nefrología, con la diálisis; neumología, con la ventilación; cirugía, con el bisturí… Sin embargo, la obligación de los cuidados paliativos es otra. Creo que esta disciplina es, en esencia, una cuestión de mirada: dónde poner el foco de la intervención. La mirada paliativa. La clave está en reconocer los beneficios de enfoques múltiples, que compensan los sesgos de cada especialidad. La visión holística. Los cuidados paliativos no emprenden ninguna guerra. Se enfoca en otros objetivos aparte de la erradicación de la enfermedad, pues al final de la vida el corazón nunca se pasará de moda^6^. La curación quedará lejos, pero desviará la mirada hacia metas más cercanas, para que nos aproveche mirarlas. Luchará, al menos, para que abran el bar de la esquina un día más^6^.

Al recordar al público corear las canciones bajo una atmósfera de despedida, me doy cuenta de que, efectivamente, el corazón no se ha pasado de moda. Nos gusta hablar de emociones, incluso de las tristes. Nos encanta preguntarnos por la identidad de los ladrones del mes de abril, aunque sea a propósito de quienes comparten su colchón con el desamparo y la humedad^9^. Siempre he percibido que las conversaciones sobre paliativos captan poderosamente la atención de casi todas las personas, dedicadas o no a la sanidad. Por eso, me fascina tanto un fenómeno que creo que todo paliativista ha palpado alguna vez, descrito con el término *Palifobia*^10^.

La *Palifobia* se refiere al miedo, duda o rechazo a implementar programas de cuidados paliativos; por parte de personal clínico, administradores y sociedad en general. Generalmente, se requiere un proceso de adaptación, en ocasiones incluso cultural, de cara a su integración^11^. La *Palifobia* surge gradualmente, a menudo con una inicial fase de negación respecto a la propia necesidad paliativa. Muchas veces se niega su pertinencia, con el argumento de que siempre se ha muerto sin una especialidad que se encargue de ello^12^. Tiene sentido, ya que un sistema tradicionalmente centrado en la curación se ha invertido poco en medir el sufrimiento de pacientes y sus familias, dejando muchas necesidades paliativas sin documentar^10^. En ausencia de datos a favor, las opiniones contrarias tienden a tener mayor peso. No obstante, hoy contamos con una creciente evidencia científica a favor de esta disciplina^13^.

La *Palifobia* es un concepto complejo, abordable desde múltiples perspectivas. Creo que sus raíces se hunden en algo aún más profundo: cómo concebimos la medicina. Mientras el arte nos ayuda a confrontar el sufrimiento, la medicina suele protegernos, incluso a costa de la verdad. Los hospitales son templos de la intervención, diseñados para combatir la mortalidad. Pero ¿qué sucede cuando la muerte es irremediable? Si la atención sanitaria fuera una canción de Sabina, los cuidados paliativos serían sus últimos versos de despedida: el adiós de una trayectoria que se extingue. Versos que, por cierto, miles de almas corean con pasión, al unísono. Por tanto, el reto no es simplemente convencer a las instituciones de financiar los cuidados paliativos, sino quizá reescribir la narrativa misma de la cultura sanitaria. No se trata solo de una cuestión de ciencia, política o economía, sino de relatos.

Aceptar los cuidados paliativos implica aceptar la mortalidad. La *Palifobia* no es solo un problema institucional: puede entenderse también como un mecanismo de defensa. Los cuidados paliativos no luchan contra la muerte, sino que le otorgan sentido. Intenta minimizar el ruido para escuchar el final^14^. Tal vez por eso incomoda a un sistema sanitario entrenado únicamente para la victoria, donde quienes cuidan reciben una escasa formación en paliativos^15^. Si las universidades instruyen exclusivamente para curar enfermedades, una especialidad enfocada en lo que ocurre cuando ya no es posible curar será, como mínimo, poco conocida. No sorprende entonces que la esencia de los cuidados paliativos no se entienda bien. Tampoco debería extrañar que persistan cierto miedo o rechazo, respuestas naturales ante lo desconocido^16^. No en vano, alguien de la altura de Kant sentenció que *el hombre no es más que lo que la educación hace de él*^17^.

Otra lectura de la *Palifobia* es entenderla como una expresión del rechazo al juicio ajeno. A los seres humanos, individual o colectivamente, no nos gusta sentirnos juzgados. Algunas especialidades podrían percibir los cuidados paliativos como una amenaza a su criterio. Comprensible: siempre se quiere lo mejor para los pacientes, y nunca agrada la acusación de agresividad terapéutica. Quizá derivar a paliativos se sienta como una rendición. Por su parte, las instituciones sanitarias podrían temer alterar modelos concebidos para la curación y el desarrollo tecnológico. Tal vez los hospitales sospechen que los cuidados paliativos cuestionan su compromiso con la compasión y la atención integral. Las facultades, a su vez, se resistirían a admitir su fracaso al no preparar para la verdad universal de que cada paciente llegará al final del camino.

Para afrontar todo esto, quizá debamos mirar más allá del ámbito de la salud, hacia las narrativas culturales que moldean nuestra percepción del sufrimiento. En una sociedad históricamente atraída por el arte que lo expresa, los cuidados paliativos podrían apoyarse en narrativas similares para una mayor resonancia social^18^. ¿Es posible resignificar esta especialidad? No como una rendición, sino como un acto de cuidado necesario e inevitable. Replantearla como una acción humana esencial, y no como mera concesión médica, podría ayudar a cerrar la brecha entre su atractivo conceptual y su ardua aplicación práctica. Sus fundamentos científicos y filosóficos son lo suficientemente sólidos como para interpelar más hondamente a una sociedad paradójicamente atraída por las emociones negativas en el arte^19^.

Demasiada divagación para una madrugada de guardia, me temo. No sé si tiene mucho sentido aquí escrito. De una cosa sí estoy convencido: la realidad supera la ficción. Los hospitales son ventanas abiertas al mundo, mucho más grandes que un escenario. A diario soy testigo de sufrimiento extremo, inefable para mí. Pacientes inmersas en un tour por su particular monte calvario, incluso a lomos de yeguas sombrías, mientras añoran lo que jamás sucedió^8^^,^^20^^,^^21^. Altas dadas a residencias en las que también habita el olvido^22^. Curas veteranos, lejos de sus años de monaguillos, prestos para varias extremaunciones^23^. Personas sentadas en una sala de espera sin esperanza, sin saber que su futuro traje de madera se plantó hace mucho^4^^,^^23^. Cada escena podría contarse con un verso distinto. Familiares que, si pudieran, no pasarían allí ninguna de las apenas quinientas noches que restan para el final^24^. Gente que, aunque preferiría estar en cualquier otro lugar, de repente comprende que al lugar donde has sido feliz no debieras tratar de volver^21^. Agitaciones nocturnas que certifican el éxodo de oscuras golondrinas^25^.

Sin embargo, encuentro consuelo al pensar que siempre nos quedará centrarnos en la persona. La humanización. Una atención holística. Cuidados paliativos. Es difícil que el fin del mundo te pille bailando cuando ya figura en la historia clínica esa nota que reza *permitir muerte natural*, pero intentaremos que suene la música^6^. Llevaremos a la cama el monigote de miga de pan a quienes la astenia ya no deja ir al Rastro a comprarlo un domingo^21^. Remendaremos sueños rotos por el suelo de ese bulevar llamado planta de hospitalización, oscura cual túnel sin tren expreso^26^^,^^27^. Mitigaremos el ruido de fondo, para que se oiga el ruido del mar^14^. Publicaremos más y mejor, para que las verdades sobre los cuidados paliativos no tengan complejos; para que no se pierdan entre cuentos de hadas^6^. Afinaremos bien dónde mirar, para que, a ambos lados de la mesa de la consulta, nos aproveche mirar lo que miramos^6^.
